# Designing antibody against highly conserved region of dengue envelope protein by *in silico* screening of scFv mutant library

**DOI:** 10.1371/journal.pone.0209576

**Published:** 2019-01-10

**Authors:** Abhishek Singh Rathore, Animesh Sarker, Rinkoo Devi Gupta

**Affiliations:** Faculty of Life Sciences and Biotechnology, South Asian University, New Delhi, India; University of Malaya, MALAYSIA

## Abstract

Dengue being one of the deadliest diseases of tropical regions, enforces to put continuous efforts for the development of vaccine and effective therapeutics. Most of the antibodies generated during dengue infection are non-neutralizing and cause antibody dependent enhancement. Hence, making a potent neutralizing antibody against all four dengue serotypes could be very effective for the treatment. However, designing a single antibody for all serotypes is difficult due to variation in protein sequences. Therefore, the objective is to identify conserved region of dengue envelope protein and then develop an antibody against that conserved region. Before advancing to the development of such an antibody, it is desirable to validate the interactions between antibody and dengue envelope protein. *In silico* analysis of such interactions provides a good platform to find out a suitable region to design and construct an antibody against it by analyzing antigen-antibody interaction before synthesizing the antibody. In this study, two highly conserved regions of dengue envelope protein were identified and an scFv was constructed against it. Both scFv and FuBc proteins were expressed in bacterial expression system and binding efficiency was analyzed by SPR analysis with *K*_*D*_ value 2.3 μM. In order to improve binding efficiency, an *in silico* scFv mutant library was created which was virtually screened for higher binding efficiency. Six mutants with high binding efficiency were selected for further analysis. The binding ability of these mutants were predicted using simulation analysis which shows these mutations were stabilizing scFv-FuBc complex.

## Introduction

Dengue is currently among world’s most prominent tropical diseases [1] and its occurrence has increased >30-fold in recent decades. There are 390 million dengue infections per year, of which 96 million manifest clinically [2]. An estimate of 3.9 billion people in 128 countries are living in areas with high risk of dengue virus infection [3]. However, there is no specific drug approved for the treatment of dengue fever. Dengue is caused by dengue virus of family Flaviviridae of (+) ss RNA virus which is mainly transmitted by female *Aedes aegypti*. There are four distinct serotypes of the dengue virus DENV-1, DENV-2, DENV-3 and DENV-4. Infection by any one of these serotypes provides life-long immunity against that serotype, but cross immunity to the other serotypes is partial. Subsequent infections by other serotypes increase the risk of developing severe case of dengue which is known as dengue haemorrhagic fever and dengue shock syndrome [4].

First dengue vaccine that has been registered for use in several endemic countries is Dengvaxia (CYD-TDV). However, there are potential risks involved with subsequent infection in areas with less exposure to dengue [5]. Research on tetravalent live-attenuated vaccines is under phase III clinical trial, and other vaccine candidates like protein subunit, DNA and purified inactivated virus are at initial stage of clinical development [6]. In dengue infection most of the circulating antibodies are non-neutralizing and are mostly directed against E protein and the prM protein. In the absence of highly specific neutralizing antibodies, non-neutralizing antibodies usually enhance the entry of any DENV into Fc receptor-bearing cells. This phenomenon is called antibody-dependent enhancement (ADE) [7] which makes DENVs unique among all the human viral infections. Nature of the antibody response to DENV is likely to play a major role in defining disease outcome. Antibodies that recognize neutralizing epitope help in virus clearance and reduced symptoms, while antibodies that recognize non-neutralizing epitopes might lead to more severe disease. Hence, there is a need of a highly specific and cross neutralizing antibody that could neutralize all four serotypes and at the same time avoid the ADE of the disease [8].

The prime target for developing an antibody is DENV envelope protein which has three structural domains DI, DII, and DIII. Domain III is present at one end of protein molecule which is involved in host cell entry. Fusion loop, present within domain DII located at the other end, provides fusion of viral membrane with the target cell endosomal membrane and help to release viral genome into the cytoplasm [9]. Efforts have been made to raise effective antibody against dengue virus. Lai, *et al*. reported that antibodies against envelope protein during infection were predominantly cross-reactive and recognize epitopes containing highly conserved residues at the fusion loop of domain II [10]. Mutation of fusion loop impairs capability of virus to infect host cells [11]. Antibody against highly conserved 20 amino acids long (250–270 amino acid) exposed surface, was reported from the infected patients indicating its antigenicity and potential vaccine development [12]. Strongly neutralizing monoclonal antibody targeting envelope protein DII has also been generated from mice immunization [13] which suggests that DENV envelope protein is a potential target. Recombinantly expressed DIII domain of DENV-3 was used to raise anti DENV-3 antibody in mice which is found to be immunogenic and can be used to elicit an immune response [14]. Specifically targeting and highly neutralizing mAbs against EDIII have been developed against sequence-unique epitope, from position 346 to 360 in the envelope protein [15]. EDIII specific antibody 4E11 has been engineered to improve affinity towards antigen on the basis of physicochemical properties of antigen antibody surface [16]. However, a linear array epitope technique was used to generate epitope specific antibody against DEIII [17]. An envelope protein dimer epitope (EDE) was created by linking two envelope proteins via disulphide bond that buries fusion loop. Antibodies raised using EDE are reported to be highly neutralizing and cross reactive [18]. Immunogenicity against different epitopes of envelope protein has also been confirmed by using monoclonal antibodies and human sera [19, 20]. These methods can help us find the neutralizing epitopes. However, none of the above have been reported to neutralize all the serotypes. Hence, the goal of our current study is to find highly conserved region among all of the four serotypes and to develop an antibody against it.

For this study, scFv antibody fragment was developed instead of whole immunoglobulin molecule which could be very effective against all serotype. scFv antibody fragment was designed using sequence of an anti-fusion loop dengue E53 Fab antibody (PDB ID. 3IXY) [21]. Molecular simulations and homology modelling are used for constructing and checking reliability of interaction between antibody fragment and its corresponding antigen. Further, binding affinity of such antibody can be enhanced by screening mutant library in the CDR regions. Subsequently, mutational studies has been performed virtually by creating single and then triple mutant libraries. In addition, six positive mutants have been selected and RMSD values have been calculated for identifying the best binding and stabilized mutant.

## Materials and methods

### Conservation analysis of dengue envelope protein

Sequences of dengue envelope protein of all four serotype were obtained using virus variation resource of NCBI. 3056 full length sequences were obtained after collapsing all the repeated sequences. These sequences were aligned using multiple sequence alignment tool of NCBI virus variation resource and analysed using Antigen variability analyser (AVANA). Regions of envelope protein with high level of conservancy were screened by setting minimum conservancy to 90% and sequence length of 8–25 amino acids. Conserved regions found in envelope protein region of sequence were further screened.

### Construction of scFv and FuBc protein sequences and homology modelling

scFv antibody was created using a reported Fab antibody from Protein data bank (PDB: 3IXY) that was highly specific for fusion loop of dengue envelope protein. V_H_ and V_L_ region of 3IXY scFv antibody was selected and joined using a (G_4_S)_3_ linker. All the six CDRs of V_H_ and V_L_ were mapped using Kabat and Chothia numbering scheme. FuBc protein was constructed on the basis of two highly conserved loops of domain II of dengue envelope protein. Whole domain II could not have been taken without incorporating domain I as they span over each other and only highly conserved regions were selected for this study, so neighbouring residues of two highly conserved loops Fusion loop and Bc loop were selected to form a stable protein molecule. This protein was named FuBc protein. Homology model of scFv was built using Swiss-model online tool. scFv x-ray crystallography structure 1QOK which shared 68.75% identity with 3IXY scFv was used as a template for homology modelling. Dengue envelope protein x-ray crystallography structure (PDB:1AON) was used as template for homology modelling of FuBc by using Discovery studio 4.0 software.

### *In vitro* binding between scFv and FuBc

To study the interaction between newly developed scFv and FuBc, SPR experiment was performed on a Biacore T200 instrument (GE Healthcare). Before going to the interaction assay both of the proteins were expressed in *E*. *coli* BL21 and purified by size exclusion chromatography. Recombinant FuBc protein was immobilized on the surface of thin gold disc of SPR instrument by amine coupling reaction. The purified scFv were applied in experimental flow cells at different concentrations going down from 10 μM to 50 ηM, as well as a non-specific protein sample was applied in control flow cells to obtain its relative binding profile. In all SPR experiments, nonspecific binding obtained in the control flow cell was substracted from the signal obtained in the experimental flow cell. For quantitative binding analysis, different concentrations of scFv protein in 100 μl of running buffer were injected at a flow rate 20 μl/min over 2 min. In between injections, the surface of the sensor chip was regenerated by injecting 2 M NaCl for 15 s at the rate of 20 μl/min. The bulk signal caused by refractive index differences between the flow buffer and the buffer containing the analytes were systematically excluded from the data-fitting process. The association constant (*k*_*a*_) and the dissociation constant (*k*_*d*_) were calculated from the association and dissociation kinetics of scFv-FuBc interaction sensorgram.

### *In silico* interaction analysis of the binding interface

Interaction analysis between scFv and FuBc protein was done using ZDock program of Discovery studio 4.0. scFv, and FuBc were defined as receptor and ligand respectively. Fusion and Bc loop were selected as the binding site to filter the poses. Poses were ranked based on ZRank score and RMSD cutoff was set at 10. ZDock results were obtained in a different window showing all the possible interaction poses and clusters. From all the clusters present on the surface of scFv protein, few clusters were filtered out on the basis of their position and interaction on the scFv molecule. All the clusters that were interacting in the CDR region of scFv antibody were filtered out. Poses among these clusters were further screened on the basis of ZDock score, ZRank score and cluster size. On the basis of these parameters best pose was found, and was further used for interaction analysis. Interaction between scFv and FuBc protein was done on the best pose found in ZDock analysis. Interaction analysis was done using Analyse Protein Interface Program of Discovery studio 4.0. The resulting data showed those amino acids which were present on scFv-FuBc interaction interface that were playing role in interaction. Different types of interactions present between the two proteins were also analysed.

### *In silico* scFv mutant library creation and screening of positive mutants

To create beneficial mutants, all the interacting residues of scFv that were involved in interaction with FuBc were selected as a target for mutation. Predict stabilizing mutation program of Discovery studio 4.0 was used to carry out saturation mutagenesis of all scFv interacting residues. All the stabilizing single mutations with negative mutation energy were selected, and were screened for best mutation possible at every position. After screening the single mutations, triple mutations were created using the combination of best single mutants. Then, the best triple mutants were screened that gave best stability with combination of three mutants.

### Homology modeling of the mutants and its interactions with FuBc

Best triple mutants were selected as a possible *in vitro* mutatable target to improve scFv-FuBc binding affinity. To validate the mutations, homology model structure of scFv was built with FuBc to observe any change in scFv structure, stability or binding affinity. Molecular overlay analysis between mutant scFvs with wild type scFv was done to measure the RMSD variation between mutant and wild type proteins. All 6 mutant structures were copied in a molecular window with the scFv wild type, and were superimposed using molecular overlay option in discovery studio. RMSD values of all mutants were calculated with reference to wild type scFv to observe the variation in scFv backbone as compared to wild type scFv.

### Simulation analysis

Finally, scFv-FuBc interaction were validated using simulation analysis which was done using the standard dynamic cascade of Discovery Studio. scFv-FuBc interaction complex was selected as simulation target. Protein was first solvated using solvation option of Discovery Studio. Solvated protein complex was then set up for standard dynamics cascade with equilibration and production simulation time set up to 20 ps and 200 ps respectively, and in advanced number of steps were set up to 4 ps. Simulation results were analysed by Analyse Trajectory Option using RMSD values. Data of RMSD variation in each simulation step was then transferred to excel. Same process was repeated with scFv, FuBc, scFv mutant1-FuBc interaction, scFv mutant2-FuBc interaction, scFv mutant7-FuBc interaction, scFv mutant8-FuBc interaction, scFv mutant12-FuBc interaction, scFv mutant15-FuBc interaction.

## Results

### Conservation analysis

**Fig 1:**
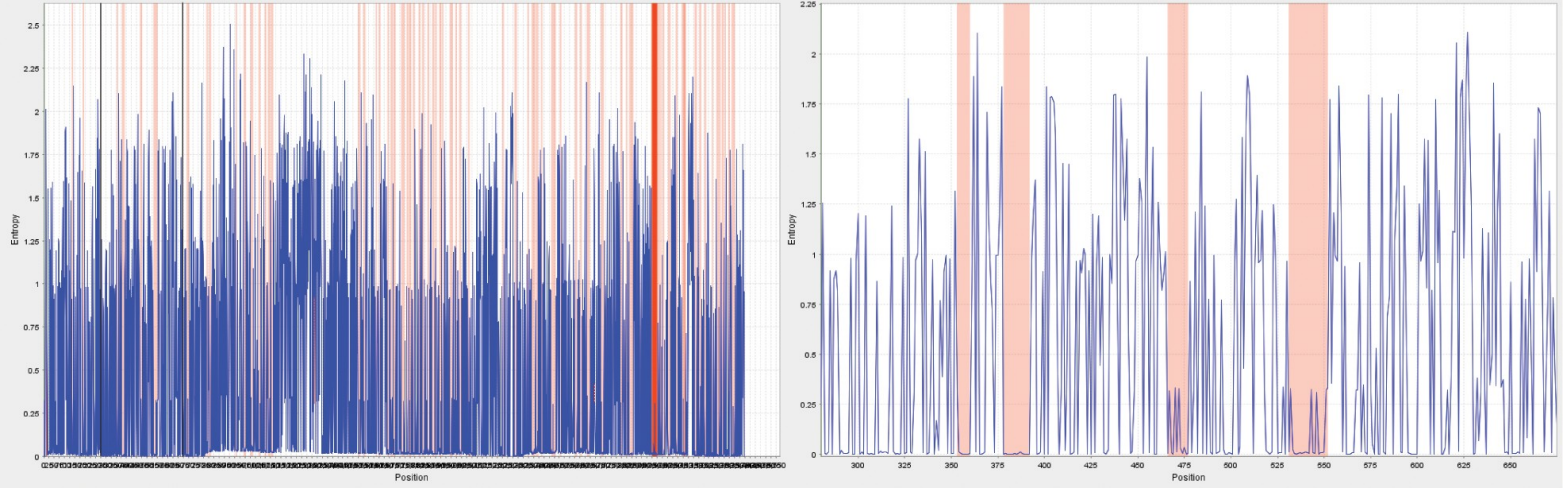
Conservation analysis of dengue envelope protein. Conservation analysis of complete sequence of dengue virus is shown in left panel, where x axis is representing sequence position and y axis is informational entropy at any particular position. Red colour represents conserved region in dengue virus genome. Dengue envelope protein is spanning from 280 to 674 position that is selectively shown in right panel.

Conservation analysis was done to find the most conserved residues in all four serotypes of dengue envelope protein. Four conserved regions were identified after aligning 3056 full length sequences of dengue envelope protein (**Fig 1**). Among these four conserved regions, two regions present in domain II of envelope protein were found to be highly conserved as well as very less informational entropy (**Table 1**). Further, it was noticed that these conserved regions were a part of fusion loop that played a major role in viral RNA internalization. These regions were named as fusion and Bc loop (FuBc), and were selected as a target for antibody binding and used for the screening of scFv antibody library.

**Table 1:** Conserved region of dengue envelope protein.

Start		End	Length	MinCons	Sequence
353	360		8	94.18%	SRCPTQGE
378	392		15	99.84%	VDRGWGNGCGLFGKG
466	477		12	93.32%	CSPRTGLDFNEM
531	552		22	93.13%	VVVLGSQEGAMHTALTGATEIQ

### Sequence retrieval and construction of scFv and FuBc

**Fig 2:**
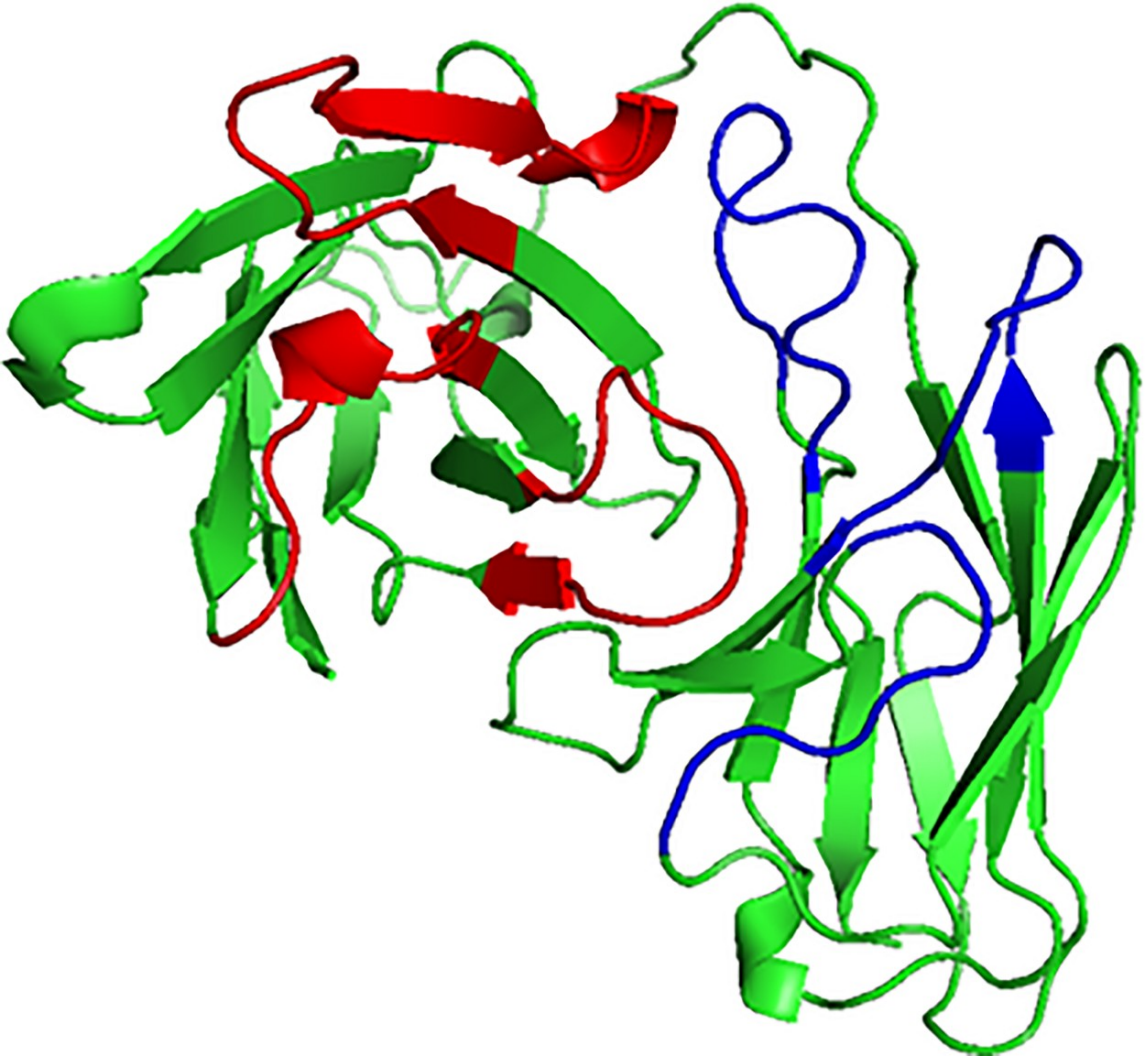
3IXY scFv structure. Cartoon structure of scFv built by homology modelling. Red and blue colours represents heavy chain CDRs and light chain CDRs respectively.

**Fig 3:**
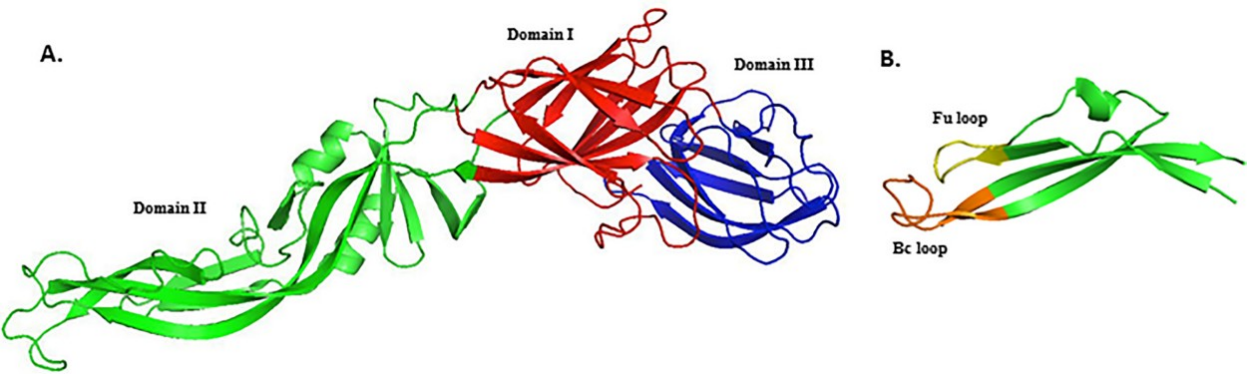
Cartoon structure of dengue envelope protein and recombinant FuBc protein. A) Structure of partial dengue envelope protein (PDB ID. 1OAN). B) Homology model of recombinant FuBc protein.

One Fab antibody (PDB:3IXY), specific against fusion loop of dengue envelope protein was retrived from PDB database [21]. V_H_ and V_L_ regions of this antibody were linked with a (G_4_S)_3_ linker to form an scFv antibody. This sequence was used as query for homology modelling. PDB:1QOK [22] structure showed highest percentage similarity to the 3IXY scFv antibody so it was selected as template to create scFv structure by homology modelling. Homology modelling of scFv sequence resulted in three models out of which the model with best DOPE score and PDF energy was selected for further analysis (**Fig 2**).Dengue envelope protein (PDB:1OAN) was selected [21]. It was a x-ray crystallographic structure of partial dengue envelope protein of DENV-2 virus shown in **Fig 3A**. FuBc sequence was created using neighbouring residues of fusion (Fu) and Bc loop (**Fig 3B**). Homology model of FuBc protein was created by using dengue envelope protein structure (PDB:1OAN) as a template. Homology modelling of FuBc sequence resulted in twenty models out of which the model with best DOPE score and PDF energy was selected for further analysis.

### Validation of scFv and FuBc interaction by Biacore assay

**Fig 4:**
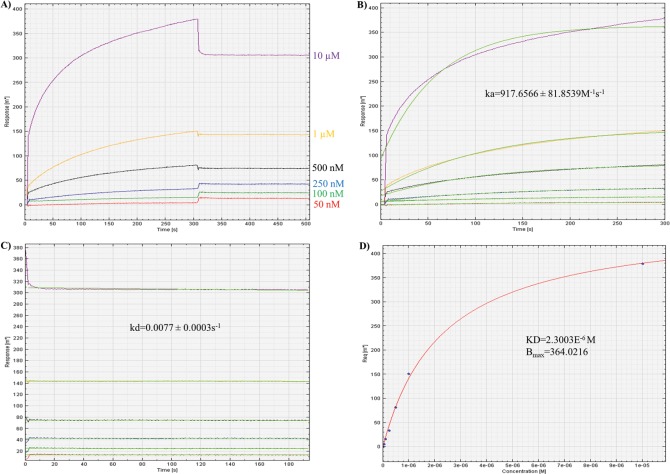
Measurment of scFv affinity to FuBc by using SPR association and dissociation kinetics. A) Sensorgram was achieved by applying scFv in mobile phase at different concentration starting from 10 μM to 50 ηM over the stationary phase of FuBC. B) Association constant (*k*_*a*_) was calculated by measuring *k*_*on*_*/k*_*off*_ kinetics from association curve. C) Dissociation constant (*k*_*d*_) was calculated by measuring *k*_*off/*_*k*_*on*_ kinetics from dissociation curve. D) Finally, scFv affinity constant (*K*_*D*_) was calculated by measuring *k*_*d*_*/k*_*a*_ kinetics from affinity curve.

To verify the *in vitro* interaction, newly developed scFv and FuBc protein were expressed and purified in soluble form. For biacore experiment, FuBC protein was immobilized on a thin gold plate of SPR instrument and scFv at different concentration were applied in mobile phase to measure their interaction as well as their affinity. From association and dissociation kinetics of scFv-FuBC interaction sensorgram, association constant, *k*_*a*_ and dissociation constant *k*_*d*_ were calculated. Finally, the affinity of scFv to recombinant FuBC protein was also calculated as 2.3003E^-6^ M by dividing *k*_*d*_ with *k*_*a*_ **(Fig 4**).

### Identification of interacting residues

**Fig 5:**
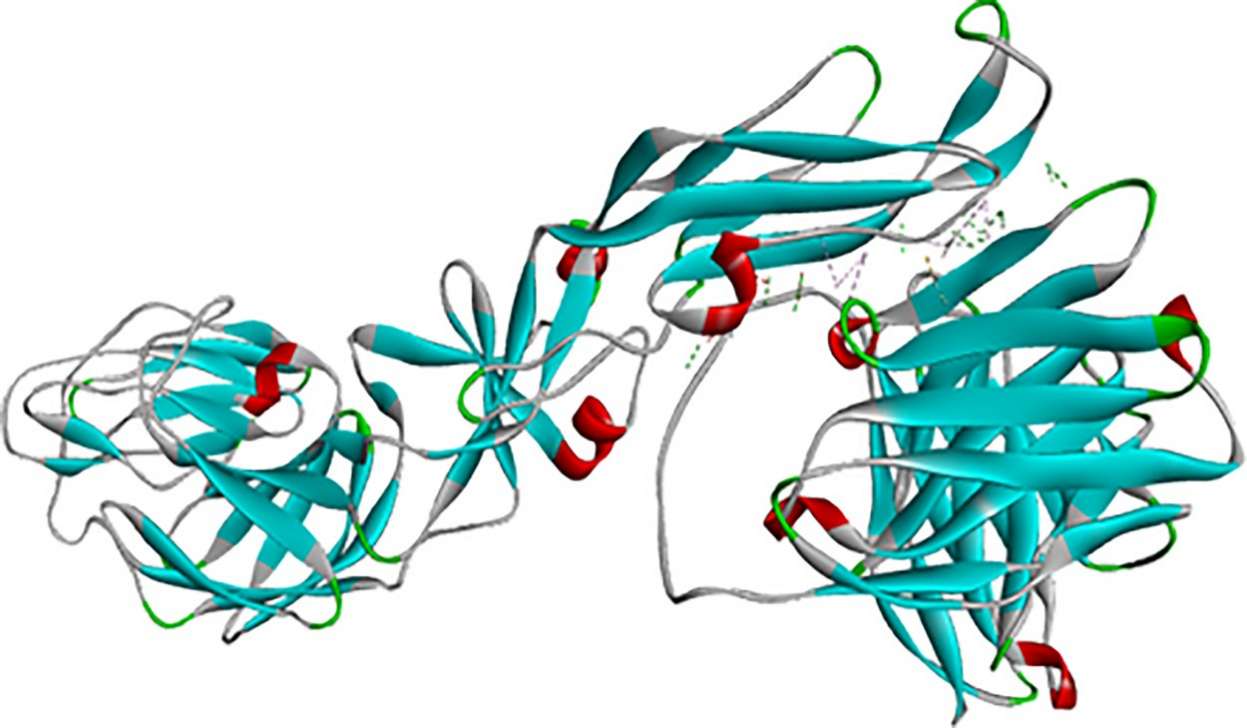
Interaction of scFv with dengue envelope protein. ZDock analysis showing interacting residues.

**Fig 6:**
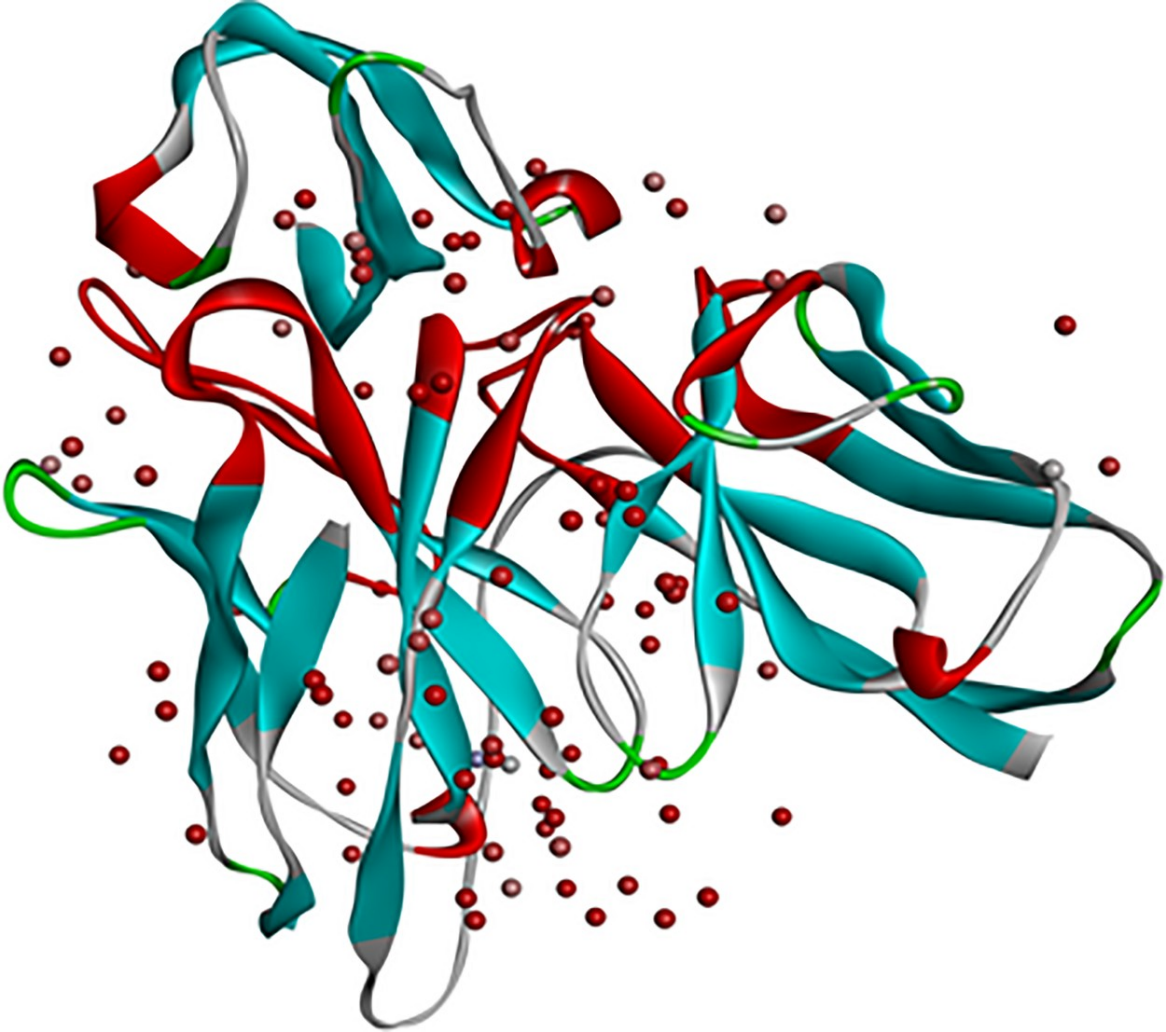
Different poses of interaction of scFv with FuBc protein. Interaction of scFv with FuBc where scFv CDRs are represented in red colour. Each docking position is represented by poses from each cluster at its cluster centre.

**Fig 7:**
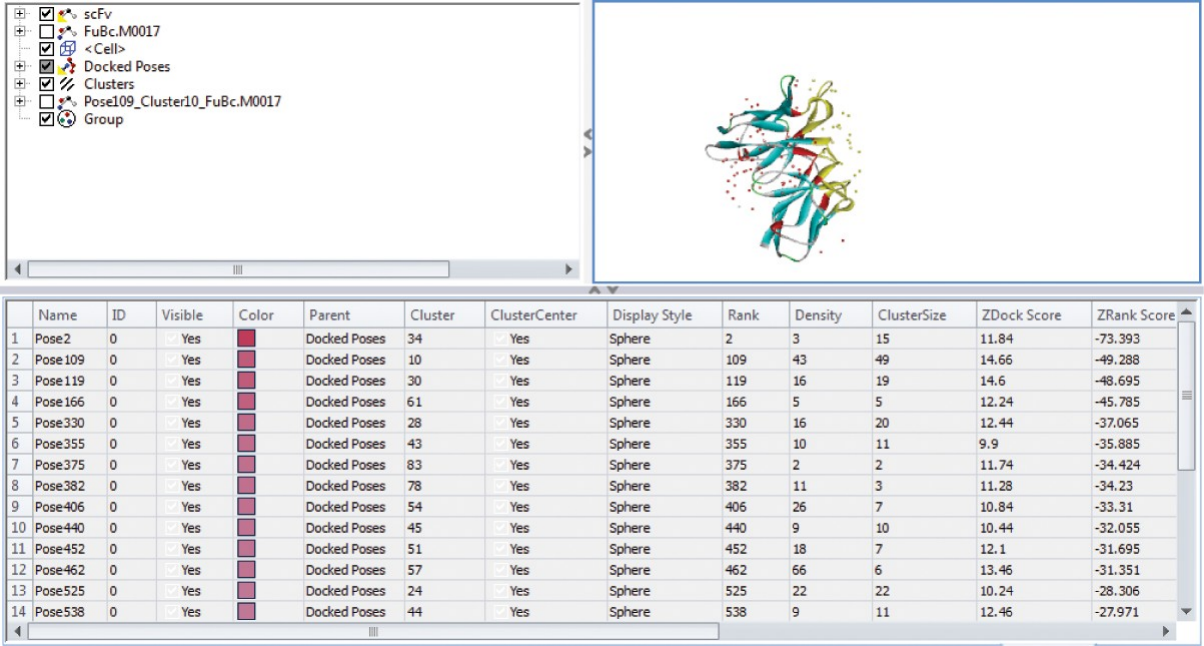
scFv with FuBc interaction pose screening. Selection of clusters with poses that are interacting with CDR region of scFv protein.

**Fig 8:**
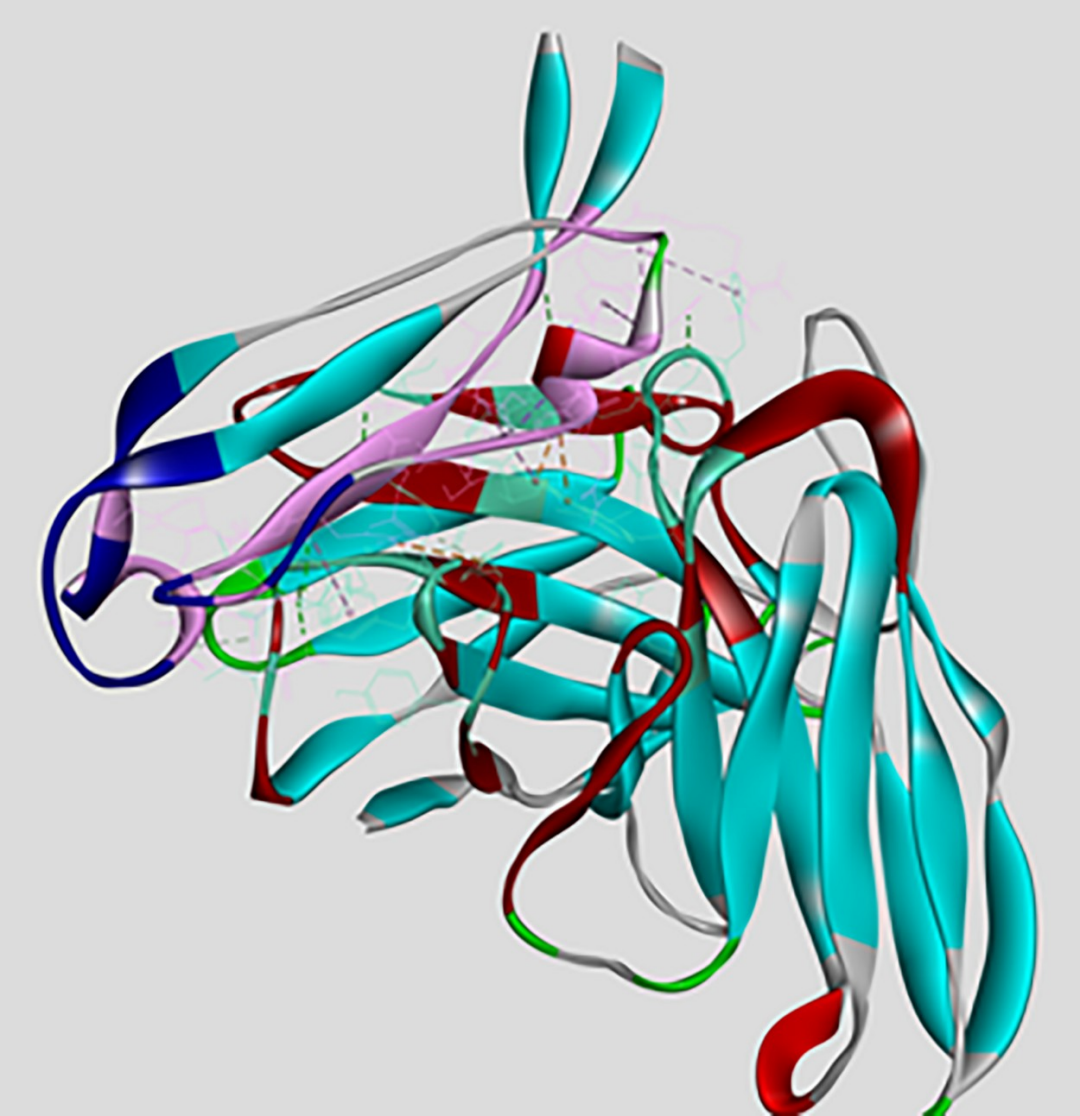
Interaction of scFv with FuBc protein. Interaction analysis between scFv and FuBc with all interacting residues in pose 109 of cluster 10. CDRs are shown in dark red colour and Fu and Bc loop are shown in dark blue colour.

**Fig 9:**
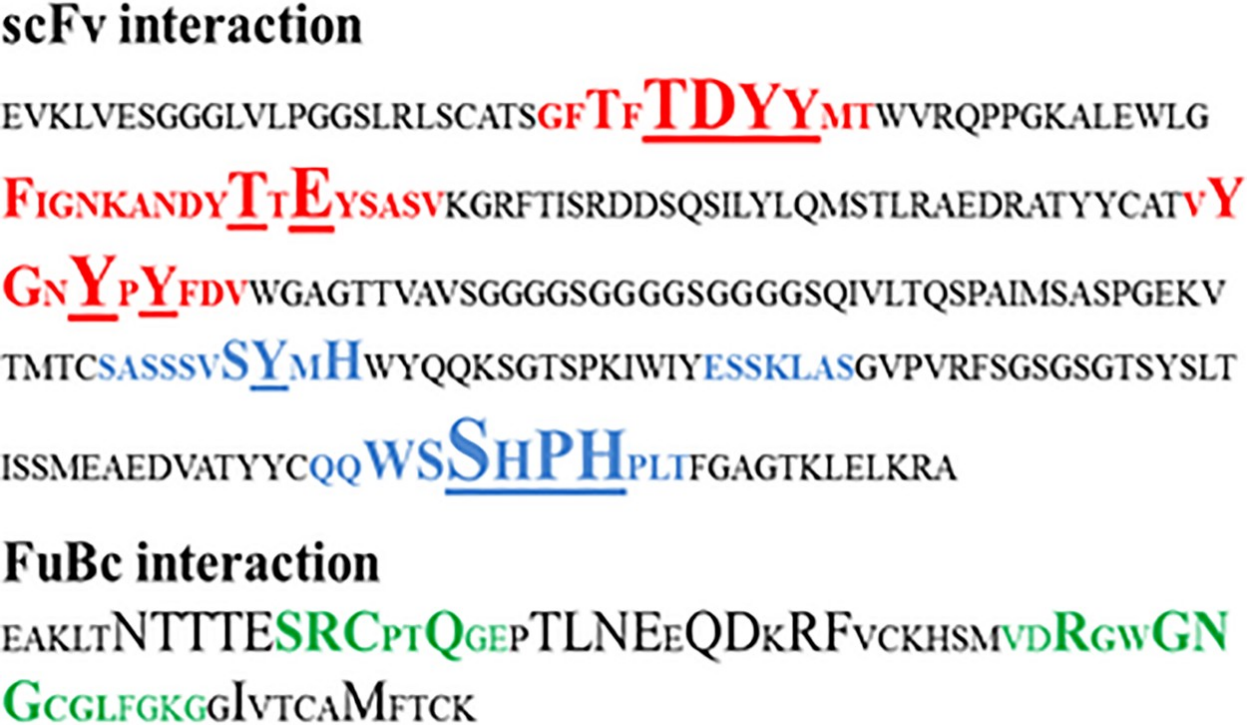
Interacting residues of scFv-FuBc interaction. scFv interacting residues are shown in bold with underline and level of interaction of each of these residues is represented by their size. Heavy chain CDRs are in red and light chain CDRs are in blue. FuBc interaction is shown in bold with bigger size. Fu and Bc loops are shown in green.

Interaction between scFv and DENV envelope protein was checked by ZDock analysis where scFv was defined as a ligand and DENV as receptor. Fusion and Bc loop region were defined as the target for binding. Residues of DENV playing role in interaction were Arg73, Pro75, Thr76, Gln77, Gly78, Glu79, Leu107, Lys110, Gly111 (**Fig 5**). These residues can be defined as hot-spots for binding of scFv antibody. For scFv-FuBc interaction analysis, 54000 poses were docked. Out of which, 2390 poses were filtered out on the basis of interaction with FuBc. These poses were present in 96 different clusters all over scFv molecule, largest of these cluster contained 198 poses. These clusters were shown by their cluster centre around scFv (**Fig 6)** and FuBc is shown in pose 109 of cluster 10. These clusters and poses were further filtered manually to find only those poses that were interacting with CDR residues of scFv antibody, and rest of the interactions were considered nonspecific (**Fig 7**). Clusters interacting with CDR residues were shown in Fig 7. Based on Z-Rank score, Z-Dock score and Cluster size, pose 109 of cluster 10 was considered to be the best pose for further interaction analysis.scFv-FuBc interaction analysis showed that 5 out of 6 CDRs were involved in interaction with FuBc molecule and highly conserved loop of FuBc had a major role in binding with scFv. This protein-protein interaction had various molecular interactions that included H-bonding, hydrophobic interaction, Pi-interaction, ionic interaction and vander waal interaction (**Fig 8**). Interacting scFv receptor residues were Thr28, Thr30, Asp31, Tyr32, Tyr33, Phe50, Thr59, Glu61, Tyr102, Gly103, Tyr105, Tyr107, Ser165, Tyr166, His168, Trp225, Ser226, Ser227, His228, Pro229 and His230. Interacting FuBc ligand residues were Asn6, Thr7, Thr8, Thr9, Glu10, Ser11, Arg12, Cys13, Gln16, Thr20, Leu21, Asn22, Glu23, Gln25, Asp26, Arg28, Phe29, Arg38, Gly41, Asn42, Gly43 and Met57. These interacting residues are shown in **Fig 9** in sequence format.

### Screening of virtually created mutant library

*In silico* mutant library was built by mutating all 21 interacting CDR residues to all 20 amino acids for selecting beneficial mutations. These mutants can be used as a target to build a small mutant library using site directed saturation mutagenesis. Predict Stabilizing Mutation Tool was used for single mutation library creation which resulted in hundreds of single mutants out of which the beneficial mutations that were positive for scFv-FuBc binding were shown in the **Table 2**. Interestingly, we noticed that multiple beneficial mutations were present at certain positions with highly negative mutation energy suggesting the stabilizing interactions. These positions can be considered as the hot-spots or the prime targets for mutations. A few of these residues are Thr30, Asp31, Ser227 and His230.

**Table 2:** All possible beneficial mutations for scFv protein that improves its binding to FuBc.

Mutation	Mutation Energy	Mutation	Mutation Energy	Mutation	Mutation Energy
THR28.GLN	-0.852045	**THR59.VAL**	**-1.06502**	**SER227.TRP**	**-3.2534**
**THR30.TRP**	**-1.90106**	THR59.ILE	-1.05671	SER227.LEU	-0.86066
THR30.PHE	-1.38101	THR59.CYS	-0.90064	SER227.ARG	-0.741184
THR30.ILE	-1.37215	THR59.PHE	-0.65846	SER227.CYS	-0.64038
THR30.LEU	-1.12846	THR59.TRP	-0.54201	SER227.MET	-0.61869
THR30.CYS	-1.08166	**GLU61.TRP**	**-1.84329**	SER227.GLN	-0.57443
THR30.VAL	-0.88388	GLU61.PHE	-1.52833	**PRO229.MET**	**-1.30307**
THR30.TYR	-0.859165	GLU61.TYR	-0.801355	**PRO229.GLN**	**-1.24191**
**ASP31.PHE**	**-3.02031**	GLU61.ARG	-0.72688	PRO229.LYS	-1.05112
ASP31.ARG	-2.34575	GLU61.MET	-0.63501	PRO229.CYS	-1.01353
ASP31.ILE	-1.81088	GLU61.GLN	-0.557085	PRO229.ALA	-0.78185
ASP31.LYS	-1.7751	**GLY103.THR**	**-0.844785**	**HIS230.PHE**	**-2.30479**
ASP31.GLN	-1.66762	GLY103.CYS	-0.757655	HIS230.TRP	-2.30271
ASP31.MET	-1.23667	**TYR105.TRP**	**-1.87079**	HIS230.TYR	-1.93659
ASP31.TYR	-1.15606	TYR105.PHE	-1.44166	HIS230.LEU	-1.84049
ASP31.LEU	-1.14876	**TYR107.TRP**	**-1.13285**	HIS230.ILE	-1.21832
ASP31.CYS	-1.09831	**SER165.TRP**	**-0.8701**	HIS230.ARG	-0.841608
ASP31.THR	-1.09109	SER165.TYR	-0.819385	HIS230.GLU	-0.696465
ASP31.ASN	-1.06624	SER165.CYS	-0.657575	HIS230.VAL	-0.62586
ASP31.HIS	-0.855045	SER165.GLN	-0.61451	HIS230.MET	-0.57906
ASP31.VAL	-0.81338	SER165.ARG	-0.564398	HIS230.SER	-0.56195
ASP31.GLU	-0.63503	**TYR166.TRP**	**-1.21901**	HIS228.ASN	-0.532915
**TYR32.TRP**	**-1.51204**	**SER226.GLN**	**-1.14458**		
TYR32.PHE	-0.52085	SER226.TYR	-1.06741		
**TYR33.GLN**	**-0.939895**	SER226.THR	-0.59387		
**TYR33.LEU**	**-0.883955**	SER226.CYS	-0.51582		

**Fig 10:**
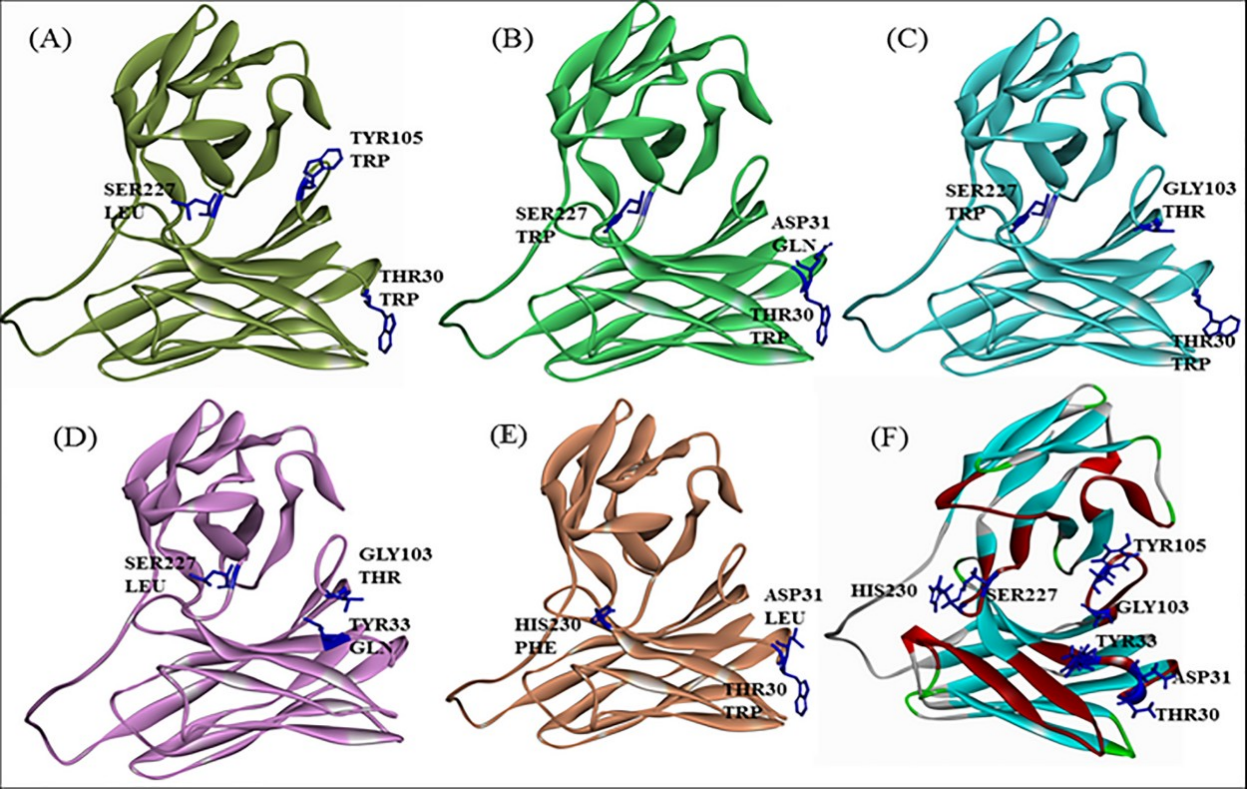
Homology model of triple mutants. Homology model of scFv mutants 1, 2, 7, 8 and 15 from table number 3 are shown as A, B, C, D and E respectively. Mutations of these mutants are shown in dark blue colour. These mutants were compared to homology model of wild type protein shown in (F).

For combinatorial mutation analysis, three different mutations were combined to give a better mutant that has better affinity than any single mutation. Top 20 of the best triple mutations were selected and on the basis of different interacting amino acid combinations, 6 mutants were further selected for homology modelling analysis (**Table 3**). Among these top 20 mutants, seven amino acid residues (Thr30, Asp31, Tyr33, Gly103, Tyr105, Ser227 and His230) were common in various triple mutations combinations. Homology modeling of the best triple mutant was Thr30-Trp, Tyr105-Trp, Ser227-Leu with a mutation energy of -9.57. Homology modelling of all the six mutants from combinatorial triple mutation library was performed and stability was checked on the basis of its DOPE score and PDF score. Structure was compared to each other as well as to the wild type where there was no structural distortion observed in CDR loop of the mutants (**Fig 10**).

**Table 3:** Best stabilizing triple mutation combination for scFv with their mutation energy.

Sr. No.	Mutation	Mutation Energy
1	THR30.TRP,TYR105.TRP,SER227.LEU	-9.57361
2	THR30.TRP, ASP31.GLN,SER227.TRP	-9.23207
3	THR30.TRP,ASP31.GLN,SER227.LEU	-8.9884
4	THR30.TRP,ASP31.LEU,SER227.LEU	-8.61851
5	THR30.TRP,ASP31.LEU,SER227.TRP	-8.576
6	THR30.TRP,ASP31.THR,SER227.LEU	-8.55091
7	THR30.TRP,GLY103.THR,SER227.TRP	-8.42681
8	TYR33.GLN,GLY103.THR,SER227.LEU	-8.3736
9	TYR33.GLN,GLY103.THR,SER227.TRP	-8.34917
10	TYR32.TRP,THR30.PHE,SER227.LEU	-8.07606
11	THR30.TRP,SER227.LEU,HIS230.TRP	-7.7884
12	TYR105.TRP,ASP31.LEU,SER227.TRP	-7.70835
13	THR30.TRP,TYR105.TRP,SER227.TRP	-7.61662
14	THR30.TRP,GLY103.THR,SER227.LEU	-7.387
15	THR30.TRP,ASP31.LEU,HIS230.PHE	-7.3348
16	THR30.TRP,TYR32.TRP,SER227.LEU	-7.20526
17	TYR105.TRP,ASP31.LEU,SER227.LEU	-7.15371
18	ASP31.LEU,SER227.LEU,HIS230.TRP	-7.08499
19	TYR32.TRP,THR30.PHE,SER227.TRP	-7.07932
20	TYR105.TRP,SER227.LEU,HIS230.TRP	-7.02525

### Characterization of selected mutants

**Fig 11:**
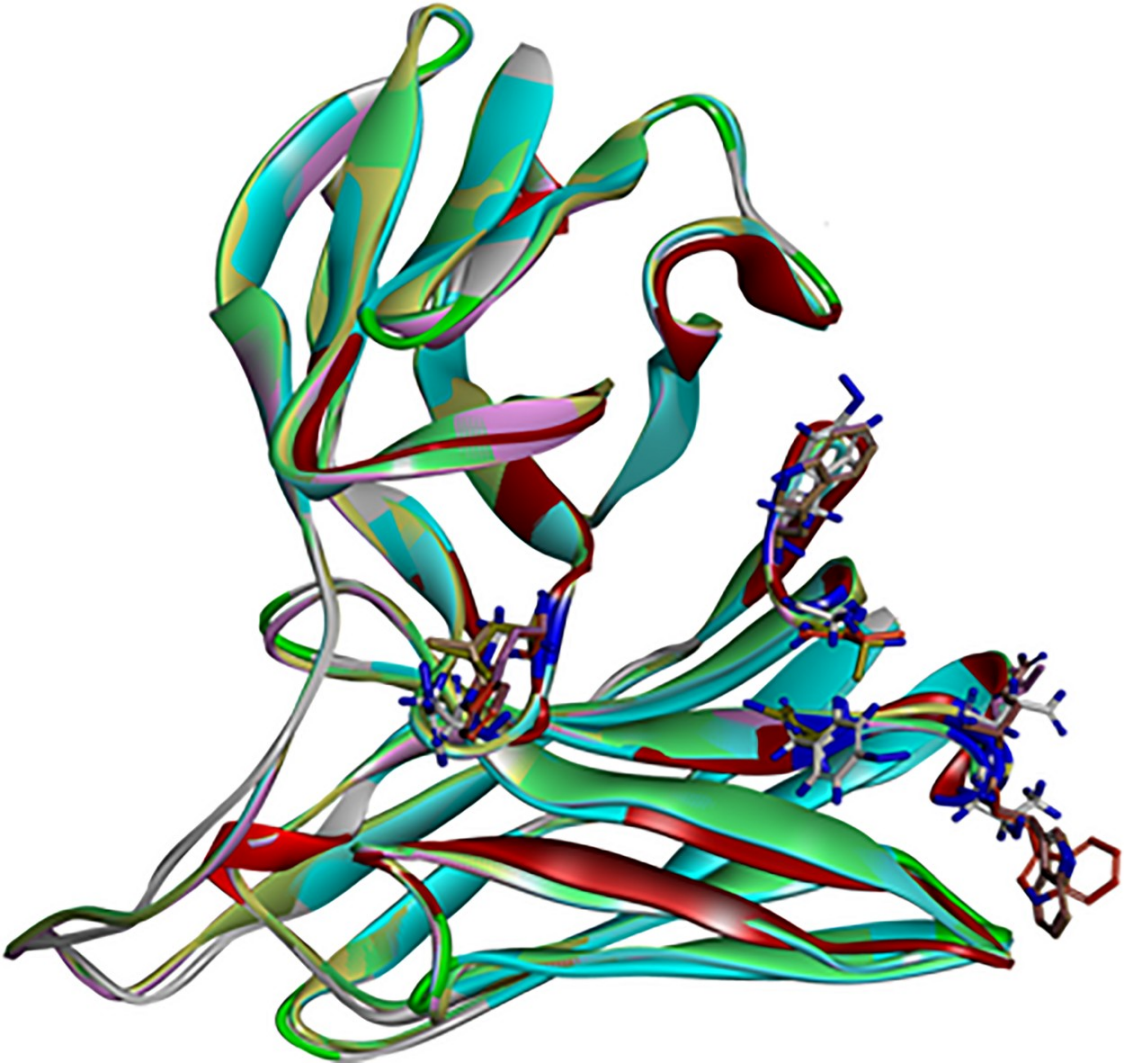
scFv molecular overlay with mutant superimposition. showing stable structure of all the mutants.

To check the variation in structure backbone due to mutations, scFv antibody was superimposed with scFv mutants homology models (**Fig 11**). RMSD values of all 6 mutants came out to be below 1.0 (**Table 4**), which signifies that all the mutants were stable and there was no mutation that is destabilizing the structure of scFv antibody.

**Table 4:** scFv molecular overlay with mutant superimposition RMSD.

Molecule	Reference	Main-chain RMSD
scFv Mutant 15	scFv 3IXY	0.756
scFv Mutant 12	scFv 3IXY	0.757
scFv Mutant 8	scFv 3IXY	0.756
scFv Mutant 7	scFv 3IXY	0.758
scFv Mutant 2	scFv 3IXY	0.757
scFv Mutant 1	scFv 3IXY	0.755
scFv 3IXY	scFv 3IXY	0.000

**Fig 12:**
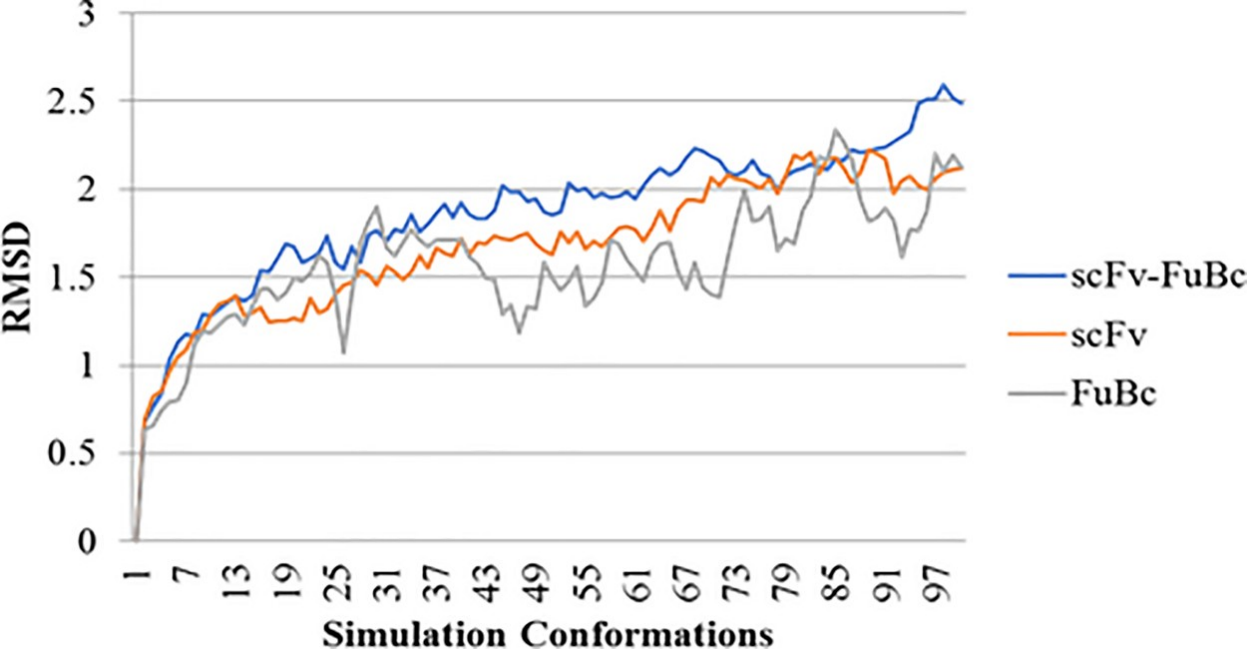
Simulation analysis of scFv FuBc interaction. RMSD value for scFv–FuBc interaction was higher than that of the individual proteins.

For the final confirmation of scFv interaction with FuBc, we proceeded with simulation analysis of scFv-FuBc interaction. To check the stability of scFv-FuBc complex RMSD values were calculated for each simulation conformation. Simulation of scFv and FuBc protein was also done separately, and RMSD of these proteins were compared to scFv-FuBc complex. It was found that RMSD value of interaction complex was higher as compared to each individual protein (**Fig 12**) which suggests that each protein was stable on its own and as compare to these protein, scFv-FuBc complex was slightly less stable.

**Fig 13:**
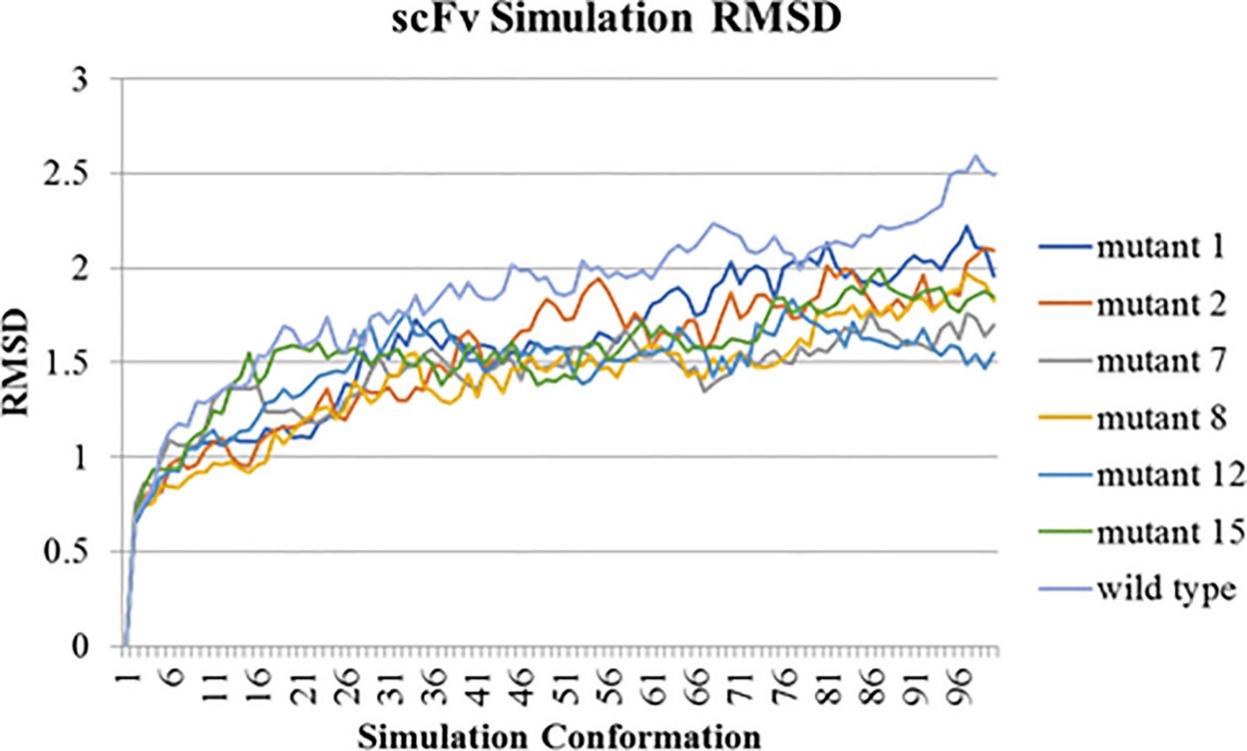
Comparison of scFv-FuBc interaction between wild type and mutant scFv. Simulation analysis of all the mutants showed less RMSD values as compare to wild type scFv.

**Fig 14:**
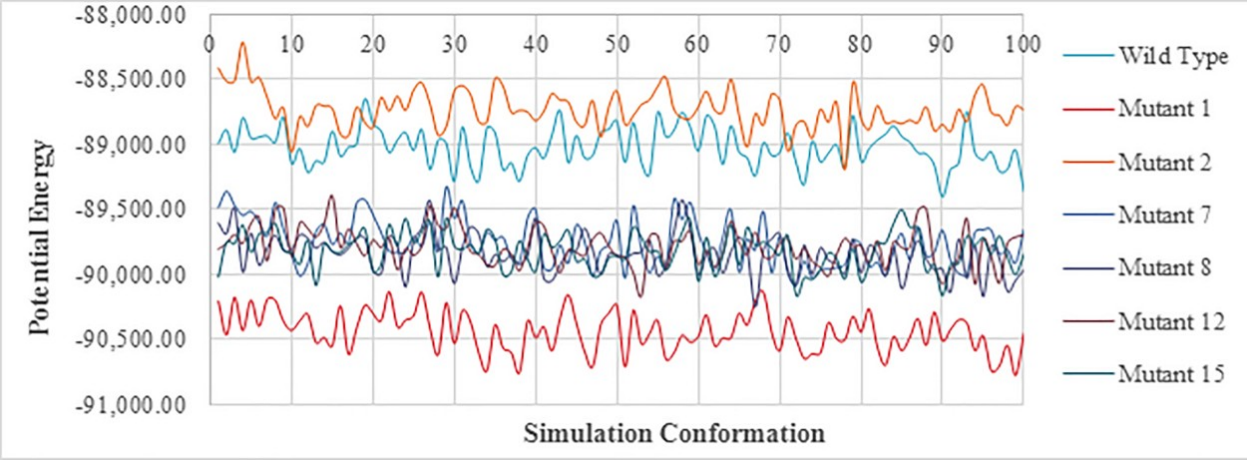
Comparison of Potential energies of scFv-FuBc interaction between wild-type and mutants. Mutant 2 showed higher potential energy as compare to wild type scFv.

To check the stability of mutants, simulation analysis was done using scFv mutant-FuBc complex. RMSD of all mutants was compared to the wild type. It was found that all six mutants were showing substantially less RMSD values as compared to the wild type (**Fig 13**). This signified these mutations were stabilizing the interaction between scFv and FuBc and leading to reduction in RMSD values. Potential energy of all the mutants was also calculated with each conformation of simulation analysis. The potential energy of scFv mutant 2 was found to be higher than that of wild type however, the remaining mutants had lower potential energy than wild type scFv (**Fig 14**).

## Discussion

Recently, structure-guided computational approaches have been utilized for several applications in the field of antibody engineering for examples, mapping of neutralizing epitope [22] and antigen-antibody interaction analysis for identification of interacting CDR residues [23]. Such analysis could be helpful in reducing the extent of work required to develop an efficient and neutralizing antibody. The envelope of dengue virus is one of the most crucial protein owing to its antigenic reactions in the host [21]. Conservation analysis of all four serotypes of DENV resulted in four peptide regions with conservation above 90%. Among these regions, the sequence between 378–392 amino acids was the most conserved (>99%). Noticeably, this region was found to be part of the fusion loop in domain II of DENV envelope protein. Another conserved region out of the remaining three, was found in another loop after fusion loop named Bc loop. Hence, these two conserved neighboring regions together (FuBc loop) were used as a target for developing cross-reactive antibody. FuBc loop was also found to be highly conserved in other viruses of *Flaviviridae* family [24]. DII of dengue envelope protein has been known to generate cross reactive antibody where fusion loop was identified as a major contributor [10]. Due to the high level of conservation of fusion loop in all serotypes of dengue, generation of cross reactive antibody can easily be assumed. However, if the binding affinity of antibody towards the conserved region can be improved, it would be able to neutralize all four serotypes of the virus.

Consequently, the sequence of scFv antibody against fusion loop of DENV was created from a pre-existing antibody by homology modelling. Previously, a novel anti-TNF scFv was constructed from human antibody frameworks and antagonistic peptides [25] suggesting a common approach to enhance the specificity and binding efficiency of scFv [26–29]. Interaction analysis between scFv and DENV envelope protein showed a stable interaction which assured that after conversion from Fab to scFv fragment, scFv antibody retained the binding property of the original antibody [30]. To further validate the interaction of scFv antibody, recombinant FuBc loop was constructed using only the conserved region of fusion and Bc loops and their neighboring residues to retain their structural integrity (**Fig 3**). When further checked for *in vitro* interaction between FuBc and scFv proteins by SPR experiment, scFv was still found interacting with fusion and Bc loop region of the FuBc protein. From the association and dissociation kinetics of scFv-FuBc interaction sensorgram, the affinity of scFv to recombinant FuBc loop was calculated (*K*_*D*_~2.3 μM; Fig 4). Though, this affinity was not sufficient in notion of therapeutic and diagnostic applications. However, the resulting scFv is undoubtedly interacting with recombinant FuBc loop and considered as a standard platform for further mutation and interaction analysis.

Affinity improvement of an antibody requires accumulation of affinity enhancing mutations in CDRs without compromising thermodynamic stability of the protein [31]. To improve the binding affinity of scFv towards FuBc, interacting amino acid residues of scFv CDRs were used as a target for mutational study. The result of saturation mutagenesis on all these scFv positions resulted in stabilizing mutations for the scFv-FuBc complex. Mutants that were destabilizing or neutral were removed and only stabilizing (i.e. beneficial) mutants were selected for this study. Total 74 beneficial mutants were found on the basis of binding energy, however these mutants were only concentrated to a few residues (**Fig 9**). These beneficial mutants were then used to build different combinations of triple mutants, and the best combinations with the lowest binding energy were screened. From the top twenty triple mutants, 6 different mutant combinations were selected for further analysis.

Homology modelling combined with molecular dynamic simulation is an efficient tool for protein structure and stability analysis [32]). So, selected scFv mutants were used to build homology models **(Fig 10)**. These protein models of scFv mutants were then used to validate the stability of scFv mutants using molecular overlay analysis. Differences in RMSD values for all mutants in reference to wild type were very low **(Table 4)**, which suggested that variation in protein backbone was minor and structural integrity of scFv molecule was also intact after the mutation. Molecular dynamic simulation was used for analysing the intricate details of scFv antibody interaction and stability [27,32–34]. In this study also, RMSD variation in scFv-FuBc complex was higher than individual scFv and FuBc conformations suggesting that scFv and FuBc proteins are very stable on its own and stabilities were more than scFv-FuBc complex. However, RMSD values of the mutant scFv-FuBc complexes were lower than RMSD of wild-type scFv-FuBc interaction complex **(Fig 13)**, and the potential energy of mutant 2 was even higher than the wild type. However, potential energy of mutant scFv 7, 8, 12 and 15 were almost equal, but lower than that of the wild-type. Mutant 1 has shown the lowest potential energy among all the mutants and was considered to be the most stable interaction **(Fig 14)**. These findings suggest that most of the mutants constructed using the triple mutant combinatorial mutant library were stabilizing scFv-FuBc interaction and improving the binding of scFv to fusion and Bc loop of dengue envelope protein.

## Conclusion

Construction of the scFv based antibody against conserved region of dengue virus envelope protein could be effective against all serotypes, and resistant to the genetic variation of the viral antigens. Conservation analysis was identified two neighbouring loops of envelope protein i.e., Fusion and Bc loop. So, these two conserved loops (FuBc protein) were selected as the target for the development of scFv antibody. The scFv antibody was created from a Fab antibody (PDB ID: 3IXY) that is reported against the fusion loop of DENV E-protein. V_H_ and V_L_ regions of the antibody were joined by a (G_4_S)_3_ linker to construct a scFv antibody. The interaction between scFv-FuBc proteins was further validated by SPR experiment. In addition, virtually the affinity of scFv was improved by mutating scFv antibody CDRs. The scFv residues interacting with the FuBc were selected as a target for mutations and the mutants with improved binding energy were selected. To improve the binding efficiency, three single beneficial mutations were stacked to make a scFv triple mutant library. Molecular overlay showed that the difference between scFv wildtype and mutants was not significant, and the mutation was not distorting the structure of scFv. The molecular dynamic simulation showed that the most of the mutants were stabilizing the scFv-FuBc interaction. RMSD and potential energy for scFv mutant 7, 8, 12 and 15 were lower than wild-type scFv. However, scFv mutant 1 showed the best potential energy and RMSD values suggesting an improved interaction. These beneficial mutations can be used to create an antibody that could be highly neutralizing against all four serotypes of dengue virus by binding to the conserved region of DENV E protein with high affinity. Hence, these engineered antibodies would be potential drug candidate for the development of a broad range neutralizing antibody against dengue envelope protein. It can also be used for preventing the viral infection by inhibiting viral entry into the host cell [35]. Moreover, the scFv sequence can be tagged with Fc domain that enables it to neutralize the virus [36]. CDRs of the scFv can also be grafted on a humanized IgG antibody molecule enabling it to deliver effector functions [37, 38].
